# Behavioral immune system activity predicts downregulation of chronic basal inflammation

**DOI:** 10.1371/journal.pone.0203961

**Published:** 2018-09-20

**Authors:** Jeffrey Gassen, Marjorie L. Prokosch, Anastasia Makhanova, Micah J. Eimerbrink, Jordon D. White, Randi P. Proffitt Leyva, Julia L. Peterman, Sylis C. Nicolas, Tania A. Reynolds, Jon K. Maner, James K. McNulty, Lisa A. Eckel, Larissa Nikonova, Jessica F. Brinkworth, Melody D. Phillips, Joel B. Mitchell, Gary W. Boehm, Sarah E. Hill

**Affiliations:** 1 Department of Psychology, Texas Christian University, Fort Worth, Texas, United States of America; 2 Department of Psychology, Tulane University, New Orleans, Louisiana, United States of America; 3 Department of Psychology, Florida State University, Tallahassee, Florida, United States of America; 4 Department of Anthropology, University of Illinois at Urbana-Champaign, Champaign, IL, United States of America; Harvard Medical School, UNITED STATES

## Abstract

Here, we present a mechanistically grounded theory detailing a novel function of the behavioral immune system (BIS), the psychological system that prompts pathogen avoidance behaviors. We propose that BIS activity allows the body to downregulate basal inflammation, preventing resultant oxidative damage to DNA and promoting longevity. Study 1 investigated the relationship between a trait measure of pathogen avoidance motivation and *in vitro* and *in vivo* proinflammatory cytokine production. Study 2 examined the relationship between this same predictor and DNA damage often associated with prolonged inflammation. Results revealed that greater trait pathogen avoidance motivation predicts a) lower levels of spontaneous (but not stimulated) proinflammatory cytokine release by peripheral blood mononuclear cells (PBMCs), b) lower plasma levels of the proinflammatory cytokine interleukin-6 (IL-6), and c) lower levels of oxidative DNA damage. Thus, the BIS may promote health by protecting the body from the deleterious effects of inflammation and oxidative stress.

## Introduction

Infectious diseases have long posed a survival threat to humans. As a result, humans have evolved numerous defenses to combat them. One such defense is the immune system. The immune system identifies pathogens within the body and responds by neutralizing and eliminating them. However, activating this system is costly. When immune cells are stimulated, they secrete a complex array of small molecular weight signaling proteins called cytokines that promote inflammation, clear/prevent infections, and heal injuries [1]. Although inflammation is critical for survival, the magnitude and duration of such events must be carefully regulated. Excessive or prolonged inflammation is known to promote or exacerbate diseases of the cardiovascular, metabolic, musculoskeletal, nervous, and immune systems [2]. Furthermore, inflammatory processes–particularly when excessive or unresolved–induce oxidative stress and reduce cellular antioxidant capacity [3–4], both of which promote aging and disease.

Given the costs of immune system activation, many organisms–including humans–also employ behavioral tactics to reduce disease burden and prevent infection, such as engaging in regular grooming [5], selectively foraging away from sites with a high pathogen load [6], and avoiding sick conspecifics [7–8]. In humans, the system that motivates pathogen avoidance behaviors is often referred to as the behavioral immune system (BIS) [9–10], or evolved pathogen avoidance system [11]. Such a system (henceforth the BIS) helps preclude the necessity of mounting a costly immune response by promoting avoidance of the source of infection in the first place. Here, we propose that–in addition to reducing infection risk and preventing the costs associated with acute immune activation–the BIS also plays an important role in minimizing basal inflammatory activity and preventing resultant oxidative stress.

The BIS is hypothesized to function by detecting disease-relevant cues in the environment (e.g., seeing someone sneeze) and activating cognitions and behaviors that minimize one’s contact with the source of infection. For example, environmental pathogen cues decrease people’s desire to be near others and reduce interest in sexual behavior [12–14]. For most people, pathogen avoidance programs such as these are only active when pathogen cues are salient [10,13]. For others, however, disease concern and the motivation to avoid pathogens are chronically high [14–16].

One of the most commonly used measures of individual differences in this construct is the Perceived Vulnerability to Disease (PVD) scale [16]. The PVD scale consists of two subscales capturing different facets of disease concern: the Perceived Infectability (PI) subscale and the Germ Aversion (GA) subscale. The PI subscale measures beliefs about one’s personal susceptibility to infectious disease (e.g., “If an illness is ‘going around,’ I will get it”). The GA subscale, on the other hand, measures one’s pathogen avoidance motivation by assessing the extent to which individuals feel discomfort in contexts connoting disease risk and are motivated to engage in behaviors that reduce pathogen transmission (e.g., “I prefer to wash my hands pretty soon after shaking someone’s hand” and “I am comfortable sharing a water bottle with a friend [reverse-scored]”). Research finds that individuals high in GA report aversion to people and objects that could act as potential pathogen vectors, even when obvious pathogen cues are absent [10,15]. For example, lower levels of extraversion are found in those with higher trait GA [16].

Many have speculated about the nature of the relationship between humans’ pathogen avoidance psychology and the activities of the immune system [17–22]. However, to date, little is known about how these variables are related to one another, if at all. Here, we propose that the BIS may function, in part, to minimize the necessity of basal inflammatory activity by decreasing pathogen contact. Specifically, we predicted that individuals who exhibit heightened pathogen avoidance motivation would have lower levels of non-targeted inflammation (i.e., inflammation that occurs in the absence of overt immune stimulation) and diminished resultant oxidative DNA damage.

We tested our hypothesis across two studies. In our first study, we examined the relationship between a trait measure of pathogen avoidance motivation (i.e., the GA subscale of the PVD scale) and indicators of non-targeted and targeted inflammatory tendencies, *in vivo* and *in vitro* (using peripheral blood mononuclear cells [PBMCs]). We predicted that people reporting greater motivation to avoid pathogen exposure would have less *in vivo* inflammatory activity and less spontaneous proinflammatory cytokine release by PBMCs in the absence of immune stimulation. However, consistent with the idea that the BIS protects the body by inhibiting non-targeted inflammation, but does not reflect low ability to mount a targeted immune response to pathogens, we predicted no differences in cytokine release by PBMCs after immune stimulation (i.e., targeted inflammation). Given that the PI subscale of the PVD measures one’s perceived susceptibility to illness, but not necessarily their motivation to avoid pathogen contact (as is captured by the GA subscale), we predicted that the PI subscale would be unrelated to basal inflammation. Nonetheless, because PI may reflect the ability of an individual’s immune system to prevent or recover from infectious illness–of which inflammation plays an important role–we also tested for the effects of PI as a predictor in all models.

In our second study, we examined the link between the same trait measure of pathogen avoidance motivation used in Study 1 and oxidative stress, a phenomenon known to result from excessive inflammation [3–4]. The body’s inflammatory response, like other metabolically costly processes, is fueled by mitochondrial respiration and the generation of adenosine triphosphate (ATP). These processes, in turn, increase reactive oxygen and nitrogen species (ROS and RNS, respectively) [23]. Although ROS/RNS serve cellular antimicrobial functions, they can also damage host DNA and contribute to diseases of aging [24–26]. Because we propose that the BIS reduces chronic basal inflammation–a key driver of ROS/RNS production–we predicted that an active BIS (i.e., greater trait pathogen avoidance motivation) would be associated with having lower levels of oxidative stress.

## Study 1

### Materials and methods

#### Participants

See S1 Table for characteristics of our sample. Sixty-two people participated (38 men, 24 women; *M*_age_ = 18.97 years, *SD* = 1.28 [range: 18–24 years old]). We chose our sample size based on the results of a pilot data collection (*N* = 16) that was completed six months prior to the data collection for the current project. We used the results of this pilot work to conduct an a priori power analysis using G*Power 3.1.5 [27] with alpha = .05 and the smallest effect size: *F*^*2*^ = .21. Results indicated that a total sample size of 62 would be needed to achieve .80 power to detect a relationship between our predictor and our weakest dependent measure. Eligibility requirements included 1) being a non-smoker, 2) being without a history of chronic medical or psychiatric disorders, 3) being non-obese [having a body mass index (BMI) below 30], 4) not taking hormonal contraceptive pills [females], 5) being free from illness for the past two weeks, 6) abstaining from steroidal and non-steroidal anti-inflammatory medications, exercise, and alcohol two days prior to the testing session, and 7) fasting the morning of the session. All women were scheduled to participate 4–7 days after the first day of their last menstrual period.

#### Procedure

Prior to data collection, the protocol for Study 1 was approved by Texas Christian University’s Institutional Review Board (approval #: 1411-117-1510AM). Written consent was obtained from all participants prior to participation. All testing sessions began at 7:30 AM after a minimum of an eight-hour fasting period. After completing questionnaires, including the target measure of trait pathogen avoidance motivation, 85 mL of blood were drawn via venipuncture into heparinized (or EDTA-containing) Vacutainer® tubes (Becton-Dickinson, Franklin Lakes, NJ). Finally, participants were thanked, debriefed, and compensated.

#### Pathogen avoidance motivation

To assess trait pathogen avoidance motivation, participants completed an established measure of this construct (Germ Aversion: GA) [16]. The GA scale is comprised of eight questions that assess participants’ tendency to experience discomfort in situations associated with germ transmission and engage in behaviors that prevent infection (α = .74). The GA scale is a subscale of the Perceived Vulnerability to Disease (PVD) scale, which also includes a second subscale, the Perceived Infectability scale (PI). This PI scale assesses the degree to which individuals perceive themselves to be vulnerable to infection (α = .93). We included both scales in our survey to help establish discriminant validity, predicting that GA, but not PI would be associated with lower basal inflammation. Both subscales were tested as separate predictors of our outcomes in each study.

#### Health and expected longevity

To assess participants’ health history, we asked them questions about (a) their experiences with infectious illnesses in the last year (e.g. “How frequently in the last year have you caught an illness from somebody else who was sick?” [scale: 1 –Never; 5 –All the Time]) and (b) their experience with more serious chronic health conditions over their lifetime (e.g., diabetes). We measured participants’ expected longevity by asking “How likely are you to be alive at ages: 20–29?, 30–39?”, and so on, through “80 and older?”. Participants indicated their responses on 7-point rating scales with the following anchors: 1 –Very Unlikely; 7 –Very Likely.

#### Immunological parameters

First, we tested for systemic inflammatory activity *in vivo* by examining plasma levels of the cytokine interleukin-6 (IL-6). Although functionally pleiotropic, IL-6 is often elevated in those with inflammatory disorders and chronically high levels contribute to diseases of aging [28–30]. Participant plasma was assayed in duplicate using commercially available high-sensitivity enzyme-linked immunosorbent assay (ELISA) kits (R&D Systems, Minneapolis, MN) with an assay range of 0.2–10 pg/mL.

Second, we isolated participants’ peripheral blood mononuclear cells (PBMCs), *in vitro*, to examine participants’ cellular inflammatory tendencies in the presence and absence of immune stimulation. PBMCs were isolated from whole blood through density gradient centrifugation in Ficoll® Paque Plus (Sigma-Aldrich, St. Louis, MO; GE Healthcare Life Sciences, Pittsburgh, PA). Cells were then washed several times in Hank’s Balanced Salt Solution (Caisson Labs, Logan, UT) to remove any extracellular signaling factors that may have been present in participants’ whole blood. The cells were next counted and plated into Falcon^TM^ 96-well tissue culture plates (Corning, Tewksbury, MA) in RPMI-1640 cell culture medium supplemented with 10% heat-inactivated fetal bovine serum, 2mM L-glutamine, 1mM sodium pyruvate, 100 U of penicillin/mL, 100μg of streptomycin/mL, and 0.25μg of amphotericin B/mL (Caisson Labs, Logan, UT) at a density of 2.5 x 10^5^ cells/well, in a 200μL volume. PBMCs were incubated for up to 3 days at 37°C, 5% CO_2_, and 100% humidity, and cytokine production was examined at 24, 48, and 72 hours after plating.

Participants’ PBMCs were plated in three testing conditions. In our target testing condition, participants’ PBMCs were plated in cell culture medium only (i.e., in sterile conditions with no immune challenge present). This was done to assess the chronic proinflammatory tendencies of participants’ cells in the absence of overt immune stimulation (i.e., spontaneous release [31]). Next, to assess the capacity of participants’ cells to respond adaptively to microbial/mitogen challenge, participants’ PBMCs were also plated with two different types of immune stimulants: lipopolysaccharide (LPS) and phytohaemagglutinin (PHA). Specifically, PBMCs were plated each with 1μg/mL of LPS serotype 026:B6, and, separately, with 5μg/mL of PHA.

We assessed release of three key proinflammatory cytokines at each of three time-points (24, 48, and 72 hours after plating) in each of three plating conditions (media-only, LPS, and PHA). The cytokines measured were interleukin (IL)-1β, IL-6, and tumor necrosis factor (TNF)-α, a trio of proinflammatory cytokines frequently produced following exposure to a wide range of pathogens *in* vivo [32]. Cell culture supernatants were assayed in duplicate using commercially available MILLIPLEX® MAP Human Cytokine Panel magnetic bead kits (EMD Millipore Corporation, Billerica, MA) with minimum detectable concentrations of 0.9 pg/mL (IL-6), 0.8 pg/mL (IL-1β), and 0.7 pg/mL (TNF-α), and an upper limit of 10,000 pg/mL for each cytokine. Plates were read using a Luminex MAGPIX® fluorescent detection system (Luminex, Austin, TX) and xPONENT® software (Version 4.2; build: 1324; Luminex, Austin, TX).

#### Alternative explanations

We assessed several other variables to help rule out alternative explanations for any associations we found between pathogen avoidance motivation and inflammatory markers. Specifically, we took measures of a series of demographic (age, ethnicity, gender, childhood socioeconomic status [SES]), biobehavioral (total adiposity, physical activity, sleep), and biosocial (stress) factors known to covary with, or directly modulate, inflammatory processes [33] and potentially impact pathogen avoidance motivation. Childhood SES was measured using an established three-item measure of this construct [34] and was also indexed by the highest educational degree earned by the participants’ mother or father. Adiposity was measured as body mass index (BMI; kg/m^2^), physical activity was measured by asking participants “How would you describe your regular level of activity or exercise?”, sleep was measured by asking participants to report on the number of hours of sleep they had gotten the night before the testing session, and stress was measured using the Perceived Stress Scale (PSS) [35].

### Results

See Data Analytic Plan in S1 Text for full data analysis plan. To examine whether trait-level differences in pathogen avoidance motivation predict lower levels of non-targeted inflammation, we conducted a series of statistical models to test the association between scores on the GA scale and *in vitro* and *in vivo* proinflammatory cytokine production.

For our *in vitro* cytokine production data, we used three-level hierarchical linear modeling (HLM software; version 6.06 [36]) to test whether pathogen avoidance motivation predicts PBMC release of IL-6, IL-1β, and TNF-α at 24 hours, 48 hours, and 72 hours post-plating, in three separate plating conditions: (a) in a resting state (spontaneous release), (b) in response to LPS, and (c) in response to PHA. HLM is appropriate for these data, as time-points of cytokine release (Level 1: 24, 48, 72 hrs.) were nested within plating condition (Level 2: media-only, LPS, PHA), which were themselves nested within individuals (Level 3). Separate models were built using each cytokine as the dependent measure. We were primarily interested in examining whether a cross-level interaction between pathogen avoidance motivation–an individual-level variable–and plating condition predicted release of each cytokine.

**Fig 1:**
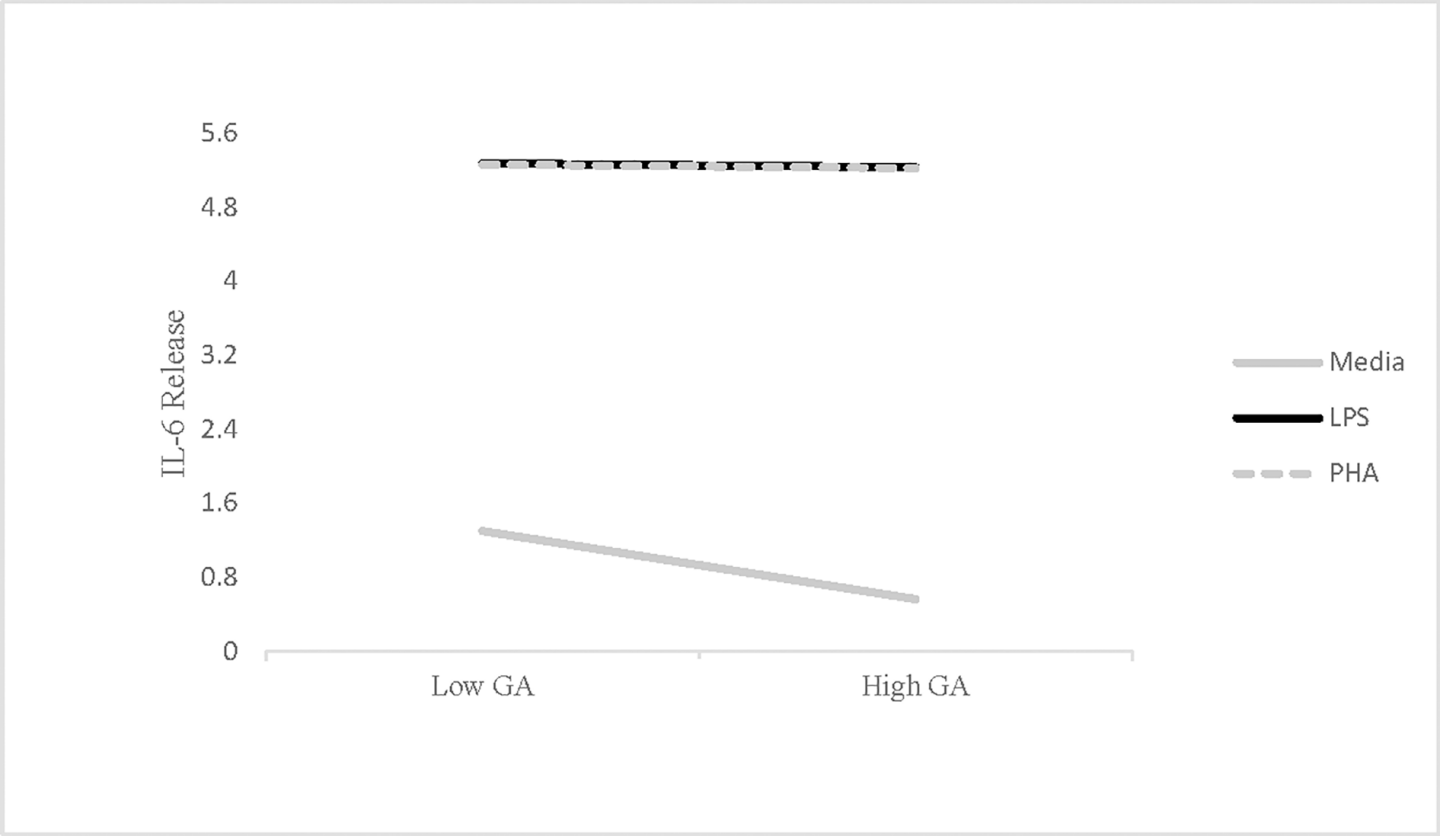
Interaction between GA and plating condition on IL-6 release. Relationship between pathogen avoidance motivation measured by germ aversion (GA) and *in vitro* IL-6 release collapsed across time-points (Study 1). High GA reflects high pathogen avoidance motivation, while higher levels of IL-6 release indicate greater inflammation. IL-6 values were natural log-transformed prior to analysis.

See Table 1 for descriptive statistics. Results for *in vitro* IL-6 release by PBMCs revealed that higher trait levels of pathogen avoidance motivation predicted lower spontaneous release of this cytokine, *B* = -.35 (*SE* = .16), *t* = -2.22, *p* = .03. Specifically, each unit increase in trait pathogen avoidance motivation predicted an average .35 unit reduction in IL-6 release in the absence of immune stimulation. As hypothesized, pathogen avoidance motivation did not predict IL-6 release in response to either type of immune stimulation (LPS: *B* = -.01, *p* = .61; PHA: *B* = -.01, *p* = .67; Fig 1).

**Table 1:** Descriptive statistics for Study 1 (*N* = 62).

Measures	*M* (*SD*)
Self-Report Measures	
Germ Aversion	3.77 (1.03)
Perceived Infectability	3.09 (1.23)
Expected Longevity	6.67 (.38)
Health History	1.18 (1.48)
Infection in Past Year	2.19 (.53)
Plasma Cytokine Levels	
Plasma IL-6	.97 (1.10)
Spontaneous Cytokine Release	
IL-1β Release	33.61 (163.45)
IL-6 Release	389.85 (1252.89)
TNF-α Release	70.21 (200.71)
LPS-Stimulated Cytokine Release	
IL-1β Release	3256.64 (3496.16)
IL-6 Release	6480.22 (2231.54)
TNF-α Release	2410.28 (1568.42)
PHA-Stimulated Cytokine Release	
IL-1β Release	1588.42 (2522.01)
IL-6 Release	6046.18 (1993.44)
TNF-α Release	2016.93 (1422.20)

*Note*. Cytokine levels shown here as raw values in pg/mL averaged across time points. All cytokine measures were log-transformed prior to analysis. IL-1β = interleukin-1 beta; IL-6 = interleukin-6; TNF-α = tumor necrosis factor alpha.

**Fig 2:**
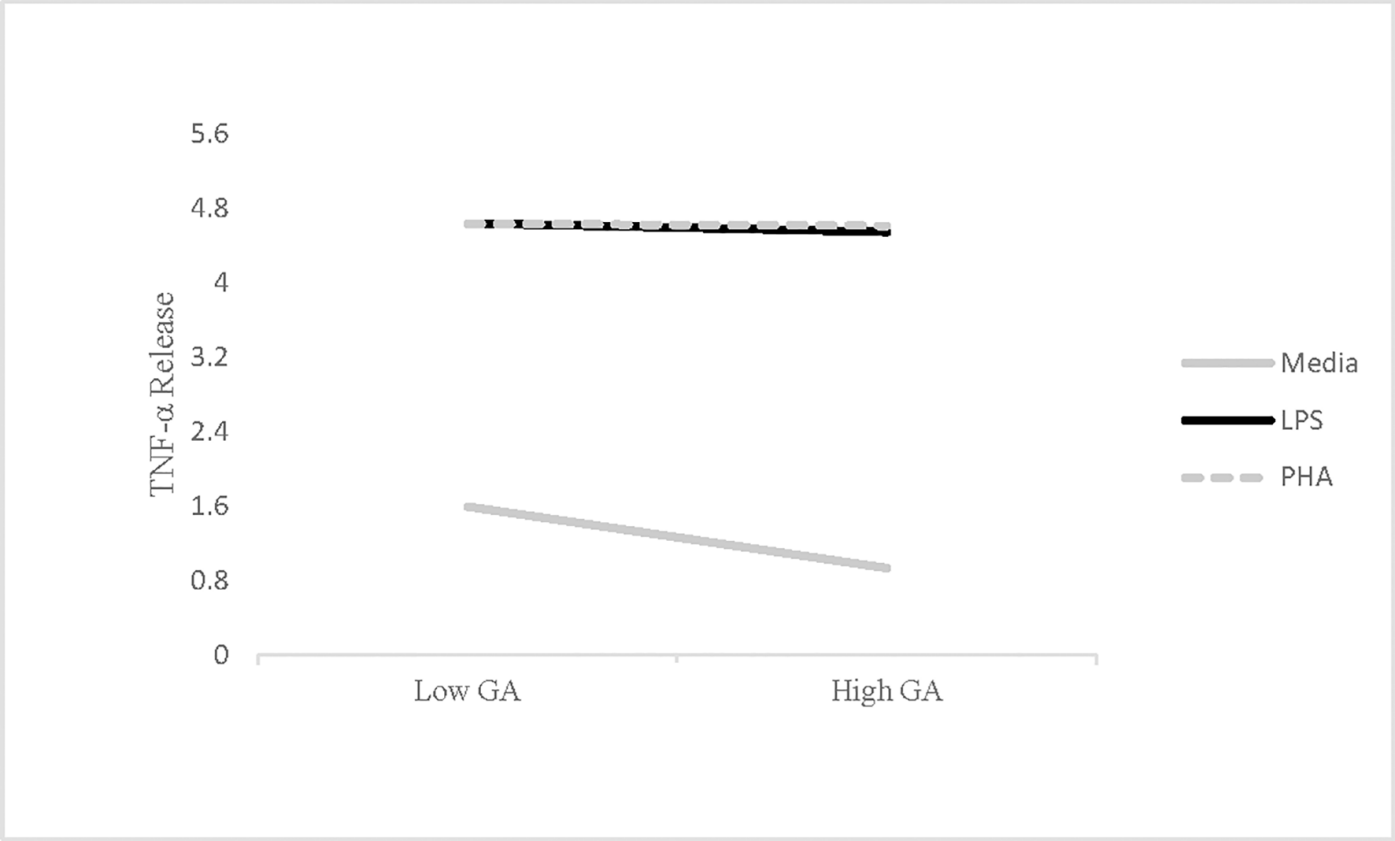
Interaction between GA and plating condition on TNF-α release. Relationship between pathogen avoidance motivation measured using the Germ Aversion (GA) scale and *in vitro* release of TNF-α collapsed across time points (Study 1). High GA means high pathogen avoidance motivation, while higher levels of TNF-α release reflect greater inflammation. TNF-α values were natural log-transformed prior to analysis.

The results for TNF-α replicated the pattern observed for IL-6 (Fig 2). Higher trait levels of pathogen avoidance motivation predicted lower levels of spontaneous TNF-α release, *B* = -.32 (*SE* = .11), *t* = -2.93, *p* = .005. Specifically, each unit increase in trait pathogen avoidance motivation predicted a .32 unit decrease in TNF-α release. As with IL-6, trait pathogen avoidance motivation did not predict TNF-α release in response to immune stimulation (LPS: *B* = -.05, *p* = .29; PHA: *B* = -.01, *p* = .74).

**Fig 3:**
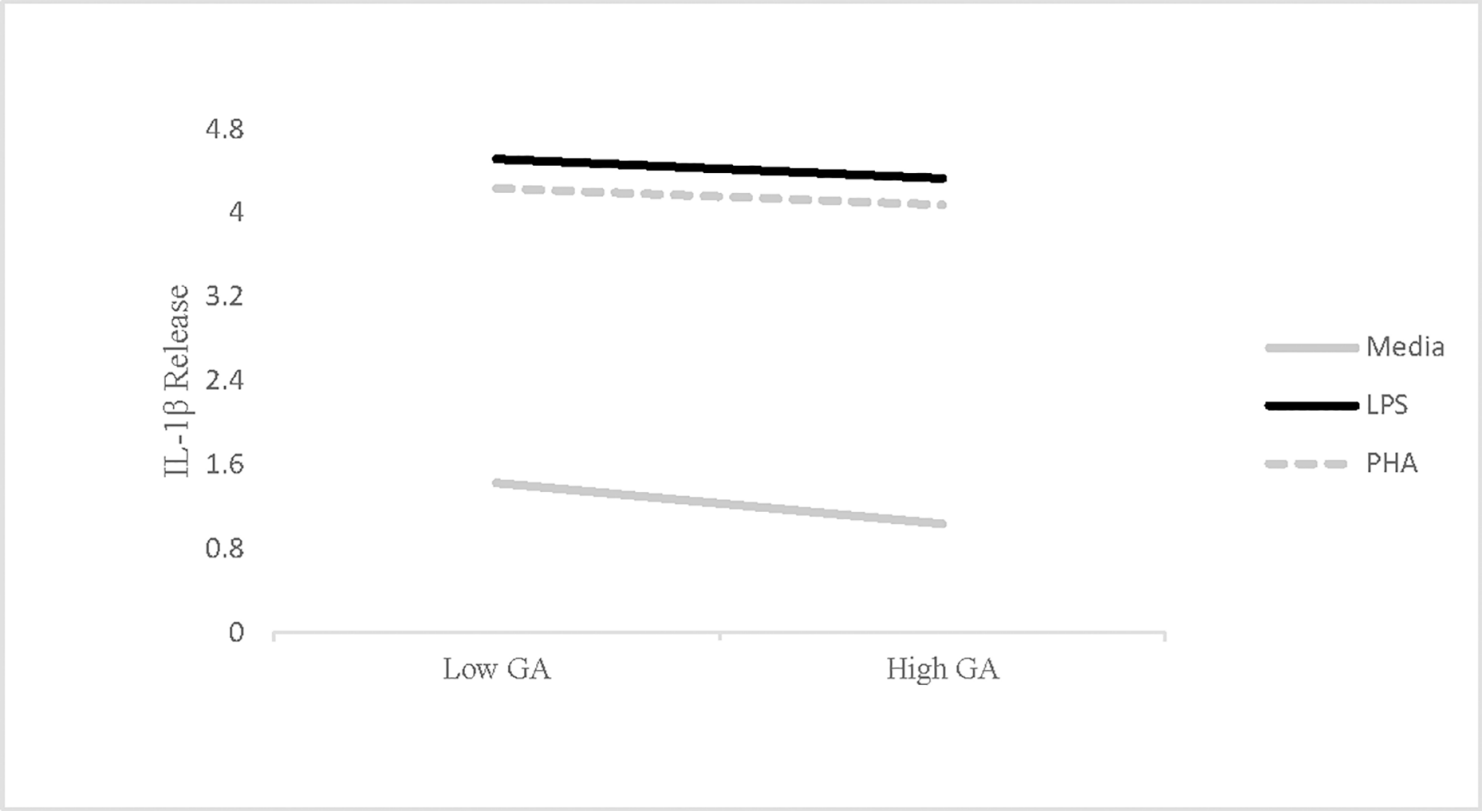
Interaction between GA and plating condition on IL-1β release. Relationship between pathogen avoidance motivation measured using the Germ Aversion (GA) scale and *in vitro* release of IL-1β collapsed across time points (Study 1). High GA represents high pathogen avoidance motivation, while higher levels of IL-1β release indicate more inflammation. IL-1β values were natural log-transformed prior to analysis.

Consistent with the findings for IL-6 and TNF-α, higher trait levels of pathogen avoidance motivation predicted lesser spontaneous IL-1β release, *B* = -.20 (*SE* = .09), *t* = -2.24, *p* = .03. Each unit increase in trait pathogen avoidance motivation predicted a .20 unit reduction in IL-1β (Fig 3). However, unlike the results observed for IL-6 and TNF-α, this slope was not significantly different than those found in the LPS (*B* = .10, *p* = .38) or PHA (*B* = .11, *p* = .33) conditions, such that pathogen avoidance motivation appeared to predict less IL-1β release both in the presence and absence of immune stimulation. Similar to the results for IL-6 and TNF-α, the negative slope of trait pathogen avoidance motivation was again steeper in the spontaneous release condition compared to either stimulated release condition. In the case of IL-1β, however, the difference in slopes did not reach statistical significance.

We then followed up our target models with two alternative sets of HLM models. First, we tested whether the results of our primary model were robust to controlling for variables known to influence inflammation, pathogen avoidance motivation, or both. Next, we examined whether effects similar to those found for trait pathogen avoidance motivation were also found for the degree to which participants perceived themselves to be susceptible to illnesses (measured using the PI subscale of the PVD scale). Results revealed that controlling for stress, sleep, age, exercise, BMI, gender, race, childhood SES, and parent education level did not change the pattern or significance of the relationships between trait levels of pathogen avoidance motivation and spontaneous proinflammatory cytokine release by PBMCs (all *p*s < .05). Further, although relationships between one’s perceived susceptibility to illness (PI) and *in vitro* cytokine release resembled those found for GA, PI did not emerge as a significant predictor of cytokine release in the media-only condition (*p*s > .09; see Alternative Models in S1 Text).

Next, to test the relationship between trait levels of pathogen avoidance motivation, *in vivo* levels of IL-6, and factors related to health and longevity, we examined bivariate correlations between GA and participants’ (a) recent experiences with infectious illnesses, (b) history of medical problems, (c) expected longevity, (d) plasma IL-6, and (e) demographic, biobehavioral, and biosocial factors known to impact each (Table 2). Results revealed that trait pathogen avoidance motivation was negatively related to *in vivo* IL-6 levels and participants’ history of medical problems, but positively correlated with expected longevity. There was no significant relationship between trait levels of pathogen avoidance motivation and participants’ experiences with infectious illnesses in the last year. Importantly, all relationships held when controlling for stress, sleep, age, exercise, BMI, gender, race, childhood SES, and parent education level.

**Table 2:** Correlations between BIS activity, Serum IL-6, demographic variables, and health.

	Germ Aversion	Plasma IL-6	Expected Longevity	Illnesses in Last Year	Medical Problems	Perceived Infectability
Gender	.14	-.06	.10	.12	-.14	.07
Race	.13	.002	-.06	.16	.17	.04
Age	-.03	-.19	.17	-.20	-.10	-.19
Childhood SES	-.01	-.05	.14	-.15	-.10	-.20
Mother Education	-.16	.05	.02	-.17	.08	-.17
Father Education	-.19	.05	.004	-.06	-.05	.07
Sleep	.06	.18	.23†	-.18	.13	-.05
Physical Activity	-.12	.13	.09	-.20	.09	-.27*
BMI	-.27*	.11	-.19	-.19	-.09	-.28*
Stress	.19	-.18	-.23	.45***	-.03	.32**
Perceived Infectability	.20	.15	-.12	.66***	.11	
History of Medical Problems	-.29*	.08	-.30*	.10		
Illnesses in Last Year	.13	.07	-.22			
Expected Longevity	.41***	-.10				
Plasma IL-6	-.30*					

*Note*. † indicates marginal significance at *p ≤* .07, indicates significance at *p ≤ .05, **p ≤ .01, and ***p ≤ .001.

**Fig 4:**
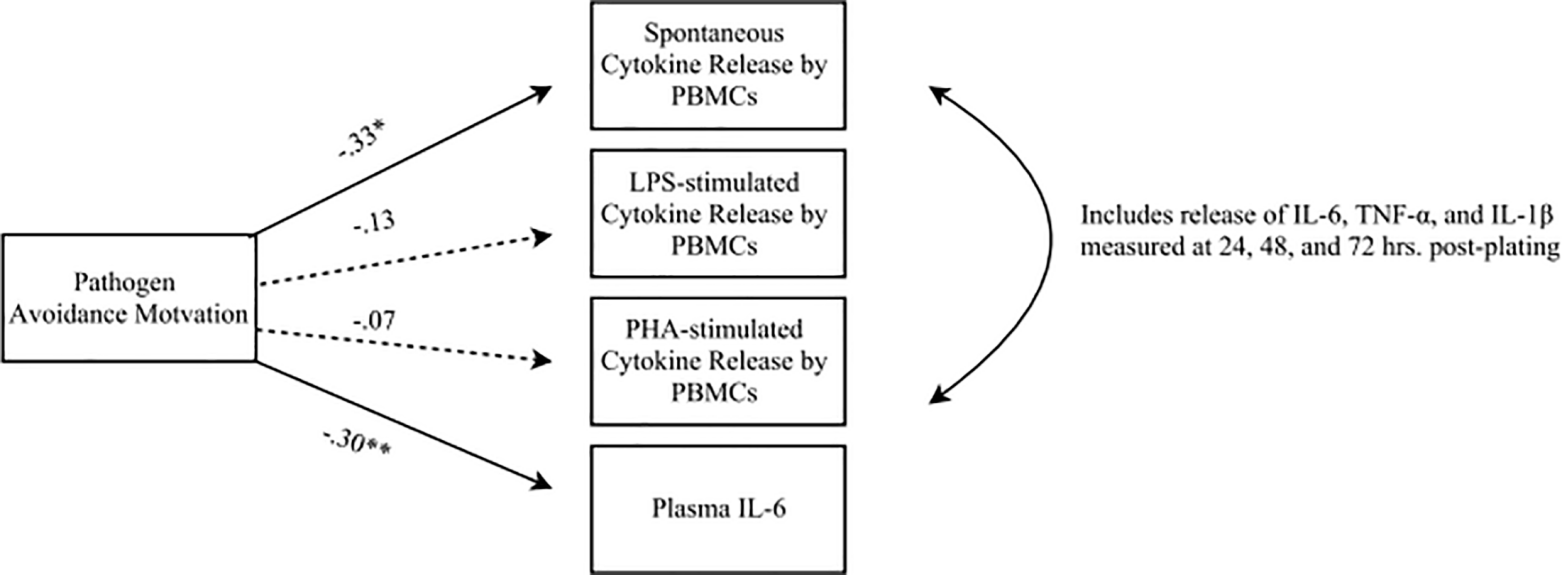
Structural equation model summarizing relationships between GA and inflammation. Structural equation model shown with standardized estimates. Dotted lines denote non-significant paths. Trait pathogen avoidance motivation measured by Germ Aversion (GA). **p < .01; *p < .05.

Finally, we combined our results into a single structural equation model (SEM; MPlus 7.4 statistical software [37]; see Fig 4 for model) by simultaneously regressing spontaneous cytokine release by PBMCs across time (cytokines presented together clustered within participant; see Data Analytic Plan in S1 Text), LPS-stimulated cytokine release by PBMCs across time, PHA-stimulated cytokine release across time, and plasma IL-6 on GA, our trait measure of pathogen avoidance motivation. Results supported the findings from the HLM models and bivariate correlations. Higher trait levels of pathogen avoidance motivation significantly predicted lower levels of spontaneous cytokine release by PBMCs, *β* = -.33, *SE* = .14, *t* = -2.34, *p* = .02, *R*^2^ = .11, as well as lower levels of plasma IL-6, *β* = -.30, *SE* = .11, *t* = -2.76, *p* = .006, *R*^2^ = .09. However, trait levels of pathogen avoidance motivation did not significantly predict stimulated cytokine release in response to either LPS, *β* = -.13, *SE* = .11, *t* = -1.28, *p* = .20, *R*^2^ = .02, or PHA, *β* = -.07, *SE* = .07, *t* = -1.07, *p* = .29, *R*^2^ = .01.

The results of our first study supported the hypothesis that BIS activity allows the body to downregulate basal inflammation. As predicted, higher trait levels of pathogen avoidance motivation were associated with lower levels of plasma IL-6, and less spontaneous (but not stimulated) release of proinflammatory cytokines by PBMCs. Additionally, results revealed that higher levels of pathogen avoidance motivation were predictive of increased perceived longevity, but not decreased illness over the last year. This result is consistent with the view that pathogen avoidance motivation may offer an alternate, but similarly effective, route to avoiding infection, without the costs of inflammatory activity.

## Study 2

In our second study, we examined whether higher trait levels of pathogen avoidance motivation were associated with less oxidative DNA damage–a frequent product of chronic inflammation [3–4]–as quantified via a key indicator of oxidative DNA base damage, 8-hydroxyguanine (8-OHdG).

### Materials and methods

#### Participants

See S2 Table for characteristics of our sample. Participants were recruited via flyers, Facebook advertising, handouts at a local medical clinic, and emails sent to couples participating in a separate longitudinal study of married couples. This yielded a total sample of 193 women (*M*_age_ = 28.56 years, *SD* = 5.59 [range: 18–40 years old]). Given the goals of the larger study from which these data were drawn, 91 of these women were pregnant. Sample size was determined based on the goals of the larger study. Participants were primarily of European descent and generally came from educated, middle-class backgrounds.

#### Procedure

Prior to data collection, the protocol for Study 2 was approved by Florida State University’s Institutional Review Board (approval #: 2014.13055). All research sessions were conducted in person and written consent was obtained from all participants prior to participation. An undergraduate female research assistant met eligible women at one of three locations: a community location (e.g., coffee shop), the participant’s home, or the research lab. Each setting had a bathroom, where urine samples could be collected privately.

Upon arriving at the appointment, participants provided informed consent and then completed a paper-based survey assessing a variety of social and individual differences, including the same trait measure of pathogen avoidance motivation used in Study 1. Next, participants provided a urine sample using sterile cups that were collected by the research assistant. Finally, participants were thanked, debriefed, and compensated.

#### Pathogen avoidance motivation

As in Study 1, trait pathogen avoidance motivation was measured using the GA scale. We again included the PI scale to test for discriminant validity. Internal consistency of each subscale was acceptable (for GA, α = .70; for PI, α = .90).

#### Oxidative stress assay

To assay oxidative stress, we followed the procedure described by Griskevicius and colleagues [38]. Immediately after research assistants returned to the lab, they pipetted the urine samples into aliquots (200uL for the 8-hydroxyguanine [8-OHdG] assay; 20uL for creatinine assay) that were then frozen at -20°C until all samples were collected. We measured 8-OHdG using the High Sensitivity 8-OHdG ELISA kit manufactured by the Japan Institute for the Control of Aging, purchased through Genox (Baltimore, MD). 8-OHdG–which is commonly assessed in urine–is one of the predominant forms of oxidative lesion to nuclear or mitochondrial DNA and an important biomarker for oxidative stress and somatic damage [39–40]. Sensitivity of this assay was approximately 0.125–10 ng/mL. To account for differences in urine concentration, all 8-OHdG values were corrected for creatinine. Creatinine assays were conducted using a colorimetric detection kit (Enzo Life Sciences; Farmingdale, NY). Creatinine values ranged from 12.84 mg/dL to 302.38 mg/dL. Following these procedures, we obtained reliable values of 8-OHdG corrected for creatinine for 165 (85%) of the participants. Specifically, we could not obtain reliable scores for 14 samples, due to low quantities or high variability between duplicates. For three additional samples, creatinine could not be determined. Finally, one participant’s sample was excluded because her oxidative stress (corrected for creatinine) was over 3 standard deviations higher than the mean (83 ng/mg creatinine).

#### Alternative explanations

We examined several other variables to help rule out alternative explanations for any relationships we found between pathogen avoidance motivation and oxidative stress. These included pregnancy status, age, childhood and current SES, number of children, and relationship status. Childhood and current SES were measured using established three-item scales [34].

### Results

First, we examined whether our key measures–oxidative stress and trait pathogen avoidance motivation (GA)–differed between pregnant and non-pregnant women. See Table 3 for descriptive statistics. No significant differences were found between pregnant and non-pregnant women in oxidative stress, *t*(133.79) = -.41, *p* = .68, or GA, *t*(163) = .93, *p* = .35, or PI, *t*(163) = -.78, *p* = .44.

**Table 3:** Descriptive statistics for Study 2 (*N* = 165).

	Pregnant (*n* = 71)	Non-Pregnant (*n* = 94)	Overall
Measures	*M* (*SD*)	*M* (*SD*)	*M* (*SD*)
Germ Aversion	4.18 (1.06)	4.05 (1.04)	4.11 (1.05)
Perceived Infectability	3.29 (1.33)	3.48 (1.32)	3.40 (1.32)
Oxidative Stress (in ng/mg) of creatinine	5.67 (3.01)	5.49 (2.49)	5.57 (2.72)

To test whether trait levels of pathogen avoidance motivation predicted lower oxidative stress, we regressed oxidative stress levels (corrected for creatinine) onto GA, PI, and pregnancy status (dummy-coded such that non-pregnant women were coded as ‘0’ and pregnant women were coded as ‘1’). PI was added into the model to examine–as we did in our first study–whether one’s perceived susceptibility to illness produced a pattern of results similar to that of trait pathogen avoidance motivation. Pregnancy status was added to the model to determine whether pregnancy moderated the relationship between GA and oxidative stress. As predicted, higher trait levels of pathogen avoidance motivation were associated with less oxidative stress, *b* = -.65, *SE* = .20, *t*(161) = -3.24, *p* = .001, 95% CI [-1.04, -0.25], *semi-partial r* = -.25. There was no main effect of PI, *b* = .10, *SE* = .16, *t*(161) = 0.66, *p* = .513, 95% CI [-0.21, 0.42], *semi-partial r* = .05, or participant pregnancy status, *b* = .28, *SE* = .42, *t*(161) = 0.66, *p* = .508, 95% CI [-0.55, 1.11], *semi-partial r* = .05, on oxidative stress. The association between GA and oxidative stress was not moderated by pregnancy status, *t*(160) = -0.38, *p* = .703. See S1 Fig for scatterplots separated by pregnancy status.

The association between GA and oxidative stress remained significant with the addition of covariates, including age, childhood SES, current SES, number of children, and relationship status, *b* = -0.69, *SE* = .20, *t*(152) = -3.44, *p* = .001, 95% CI [-1.08, -0.29], *semi-partial r* = -.26. There was a trend for age to positively predict oxidative stress levels, but it did not reach significance, *b* = .08, *SE* = .05, *t*(152) = 1.73, *p* = .086, 95% CI [-0.01, 0.17], *semi-partial r* = .13. Notably, women who reported having more biological children had lower oxidative stress than women who had fewer biological children, *b* = -.56, *SE* = .26, *t*(152) = -2.15, *p* = .033, 95% CI [-1.08, -0.05], *semi-partial r* = -.17. Neither childhood SES, current SES, or relationship status was associated with oxidative stress (all *p*’s > .25).

## Discussion

These studies provide evidence of a novel benefit afforded by the BIS. Specifically, we showed that pathogen avoidance motivation may promote health and longevity by allowing for lower levels of non-targeted inflammation without an increase in infection risk. In Study 1, people with higher trait levels of pathogen avoidance motivation had lower plasma IL-6 (*in vivo* measure) and less spontaneous *in vitro* proinflammatory cytokine release (i.e., IL-1β, IL-6, and TNF-α). In the case of IL-1β, we also found less release across immune stimulation contexts. Further, trait levels of pathogen avoidance motivation predicted neither a) self-reported history of infectious illnesses nor b) IL-6 and TNF-α release in response to immune stimulation, suggesting that diminished levels of inflammatory activity do not come at the expense of increased vulnerability to infection.

These results also provide preliminary evidence that the BIS allows for infection avoidance without the physiological costs associated with elevated basal inflammation. Indeed, pathogen avoidance motivation was *negatively* related to chronic disease history (e.g., heart disease and diabetes) and positively associated with expected longevity. The potential longevity-promoting features of the BIS were echoed in the results of Study 2, which found a link between trait pathogen avoidance motivation and protection from DNA damage. Participants more motivated to avoid pathogens had less oxidative stress, suggesting that the BIS may play a key role in minimizing chronic exposure to inflammation and associated somatic damage. Taken together, these results support the hypothesis that the BIS works alongside the physiological immune system to promote health.

### Limitations and future directions

In considering these findings, one must take into account several key limitations. For example, measuring markers of immune function or oxidative stress in human participants requires easily and non-invasively obtained measures, usually allowing for collection of only small amounts of biological samples. Thus, we measured a limited number of relevant immunological endpoints. Future work might extend the results of the current research by examining relationships between pathogen avoidance motivation and additional domains of immune function, such as markers of adaptive immunity (e.g., antibody production). Further, the majority of participants in our study were college students who have limited contact with pathogens in their environment. Although our results suggest that pathogen avoidance behaviors lead to lower basal inflammation in the context of a relatively sanitary college campus, such a link may not be found when an individual is unable to control pathogen exposure behaviorally. Moreover, in an environment where pathogen exposure is not controllable, downregulating inflammatory activity–which generally increases vulnerability to infection–may even prove dangerous. Future work is needed to determine the role one’s control over pathogen exposure plays in regulating trade-offs between BIS activity and the physiological immune system.

It should also be noted that because measures of oxidative stress and inflammation were obtained in separate studies, we are unable to definitively determine whether the lower levels of oxidative stress found in participants with higher pathogen avoidance motivation (compared to those with lower pathogen avoidance motivation; Study 2) were due to lower levels of basal inflammation in these individuals (Study 1). However, such an interpretation is consistent with the pattern of our results, as well as with previous research which finds that inflammation is a key driver of oxidative stress [3–4]. Future work is needed to address this limitation and extend the findings of the current research. A prospective study–in which inflammation, oxidative stress, and health outcomes are tracked over time in a group of individuals with high and low pathogen avoidance motivation–seems well-suited for this purpose. Such a study might also lend insight into the biological relevance of the differences in basal inflammation and oxidative stress found in the current research.

The present findings also contribute more generally to research which suggests that unstimulated (i.e., spontaneous) *in vitro* cytokine release is an important measure to consider for those exploring relationships between inflammation, psychology, and health. Previous research has found, for example, that unstimulated proinflammatory cytokine release by PBMCs is a strong predictor of the magnitude of one’s reported symptoms during an illness [41]. Others find that post-traumatic stress disorder is associated with greater unstimulated–but not stimulated–release of proinflammatory cytokines by PBMCs [42]. Although these and other lines of research have demonstrated the utility of using unstimulated *in vitro* proinflammatory cytokine release as a measure of basal inflammatory activity [31], relationships between unstimulated cytokine release and other key variables are often left unexamined in many studies. Instead, unstimulated cytokine release values are either (a) not measured [43], (b) only controlled for [44], or (c) are subtracted from stimulated cytokine release values in order to estimate a stimulated release change score [45]. Although the latter two techniques may provide useful indices of a population of cells’ (e.g., PBMCs’) reactivity to overt immunological stimulation, failure to also consider unstimulated cytokine release as an individual variable may lead to the loss of critical information about the functioning of one’s cells at baseline. Thus, one’s research question should be carefully considered before a data analysis plan for *in vitro* cytokine release data is determined.

Taken together, this set of findings provides important new data that buttress the functional importance of the BIS in managing one’s risk of infection and one’s general health. Indeed, in addition to the more obvious roles it plays in coordinating cognitions and behaviors that prevent exposure to pathogens, these new data indicate a novel and important role for the BIS in potentially obviating the need for elevated basal levels of inflammation *without* increasing the risk of infection. Accordingly, the BIS may play a role in helping curb inflammation’s costly metabolic toll and oxidative sequelae.

## Supporting information

S1 Text Additional data information for Study 1. This file contains additional information on the data analytic plan for Study 1 and extended reporting of the results for Study 1.
(DOCX)

S1 Table Characteristics of the sample for Study 1 (*N* = 62). All participants were non-smokers and came in fasting and healthy (i.e., no illnesses reported two weeks prior to the testing session). Participants were instructed not to consume alcohol, exercise, or take anti-inflammatory medications for two days prior to their testing session. All women in the sample were non-pregnant and not on hormonal contraceptives. Women’s sessions all took place 4–7 days after the start of their menstrual cycles.
(DOCX)

S2 Table Characteristics of the sample for Study 2 (*N* = 193). (DOCX)

S1 Fig **Associations between GA and oxidative stress in Study 2 for control women (panel A) and pregnant women (panel B).** Pregnancy status did not moderate the significant relationship between trait pathogen avoidance motivation and oxidative stress.
(DOCX)

S1 Data Data from Study 1. These data are also available on the Open Science Framework using DOI 10.17605/OSF.IO/2KQM5.
(XLS)

S2 Data Data from Study 2. These data are also available on the Open Science Framework using DOI 10.17605/OSF.IO/2KQM5.
(XLSX)
