# Supplementary material for: Behavioral immune system activity predicts downregulation of chronic basal inflammation
Source: PLoS One. 2018 Sep 20;13(9):e0203961. doi: 10.1371/journal.pone.0203961 (PMC6147464; doi:10.1371/journal.pone.0203961)
Supplement: S1 Text — This file contains additional information on the data analytic plan for Study 1 and extended reporting of the results for Study 1. (DOCX) [file pone.0203961.s001.docx]

Supporting Information for

Behavioral Immune System Activity Predicts Downregulation of Chronic Basal Inflammation

Jeffrey Gassen, Marjorie L. Prokosch, Anastasia Makhanova, Micah Eimerbrink, Jeffrey Gassen, Jordon D. White, Randi P. Proffitt-Leyva, Julia L. Peterman, Sylis C. A. Nicolas, Tania A. Reynolds, Jon K. Maner, James K. McNulty, Lisa A. Eckel, Larissa Nikonova, Jessica F. Brinkworth, Melody Phillips, Joel Mitchell, Gary W. Boehm, and Sarah E. Hill

Correspondence to: j.gassen@tcu.edu

Study 1

Data Analytic Plan

Analyses of the psychometric characteristics of our measures prior to analysis revealed that the target predictors and outcomes were distributed normally, with the following exceptions: (a) the variable measuring the number of serious health conditions the participant had experienced and, (b) all cytokine measures (both *in vitro* and *in vivo*) were positively skewed. Per convention, a natural-log transformation was therefore applied to each of these variables prior to analysis to normalize the skewed distribution [1]. Missing data for all hierarchical models were accounted for using casewise deletion [2].

**Hierarchical linear models for *in vitro* cytokine release**

A separate hierarchical model was generated using each cytokine as an outcome (IL-6, TNF-α, and IL-1β; HLM software; version 6.06) [2]. For each model, the linear slope of time was entered into the model grand-mean centered at Level 1. Comparisons between the three plating conditions: media-only, LPS, and PHA, were entered at level 2 as random effects (categorical; dummy-coded). First, the media condition was entered into the model coded as ‘0’ for both dummy variables to make comparisons between cytokine release in each mitogen condition and release in the absence of stimulation. We then created and entered additional dummy-coded variables to make the final comparison between LPS and PHA, and to calculate simple slopes within each plating condition. GA was grand-mean centered and entered as a random level 3 predictor of the intercept and slope, allowing us to test both for interactions between pathogen avoidance motivation and plating condition on cytokine release, as well as between pathogen avoidance motivation and the estimated change of cytokine release over time.

**Structural equation modeling of all cytokine data**

After estimating the hierarchical models and analyzing bivariate correlations between trait levels of pathogen avoidance motivation, plasma IL-6, and health, we used structural equation modeling (SEM; MPlus 7.4 statistical software) [3] to combine both the *in vitro* and *in vivo* cytokine data into a single statistical model to aid interpretation of the results. Because spontaneous and stimulated cytokine release *in vitro* contained a hierarchical structure with three time points nested within each participant for the three cytokines (IL-6, IL-1β, and TNF-α), we used the TYPE = COMPLEX command, which allows for specifying each individual as a cluster to account for non-independence in the data. For this model, spontaneous release of all three cytokines across time was presented as a single factor and stimulated cytokine release was presented as a separate single factor for each mitogen (LPS and PHA). For the final model, plasma IL-6, spontaneous cytokine release, LPS-stimulated cytokine release, and PHA-stimulated cytokine release were simultaneously regressed on trait levels of pathogen avoidance motivation (see Figure 4 in main text for final model).

**Hierarchical Linear Modeling Results**

**Pathogen avoidance motivation and IL-6**

The results of the final model revealed significant main effects of plating condition at level 2, indicating that IL-6 release was higher in both the LPS condition (*B* = 3.05 (*SE* = .14), *t* = 22.35, *p* < .001) and PHA condition (*B* = 3.03 (*SE* = .14), *t* = 21.78, *p* < .001) when compared to the media only condition (by 3.05 units and 3.03 units, respectively). We found no significant differences in IL-6 release between responses to LPS and PHA (*B* = -.03*, p* = .24). The slope of time was significant (*B* = -.002 (*SE* = .001), *t* = -3.33, *p* < .001), indicating that across plating conditions, IL-6 release declined over time. No significant interaction was found between GA and the slope of time (*B* = -.00, *p* = .74).

These results were qualified by a significant two-way, cross-level interaction between plating condition and trait pathogen avoidance motivation, which revealed significant differences in the slope of trait pathogen avoidance motivation between the media only condition compared to both LPS (*B* = .34 (*SE* = .15), *t* = 2.29, *p* = .03) and PHA (*B* = .34 (*SE* = .15), *t* = 2.28, *p* = .03). No such differences were found between the two mitogen conditions (*B* = .004*, p* = .87). Simple slopes tests revealed a significant negative slope of trait pathogen avoidance motivation in predicting IL-6 release for the media only condition (*B* = -.35 (*SE* = .16), *t* = -2.22, *p* = .03). Specifically, each unit increase in trait levels of pathogen avoidance motivation predicted an average .35 unit reduction in IL-6 release across time. The slope of trait pathogen avoidance motivation did not significantly predict IL-6 release in either the LPS condition (*B* = -.01, *p* = .61) or the PHA condition (*B* = -.01, *p* = .67).

These results suggest that pathogen avoidance motivation predicted less release of IL-6 in the absence of stimulation, but had no impact on IL-6 release in response to an immune stimulus.

**Pathogen avoidance motivation and TNF-α**

Results revealed a significant main effect of plating condition on TNF-α release, showing that compared to the media only condition, TNF-α release was 2.28 units higher in response to stimulation with LPS (*B* = 2.28 (*SE* = .11), *t* = 21.44, *p* < .001) and 2.19 units higher in response to stimulation with PHA (*B* = 2.19 (*SE* = .11), *t* = 20.45, *p* <.001). TNF-α release was also higher in the LPS condition compared to the PHA condition by .09 units (*B* = .09 (*SE* = .02), *t* = 5.01, *p* < .001). The significant slope of plating time revealed that TNF-α release also decreased with time (*B* = -.01 (*SE* = .001), *t* = -8.85, *p* < .001). Trait levels of pathogen avoidance motivation did not significantly interact with the slope of time (*B* = .00, *p* = .91).

These main effects were also qualified by a significant two-way, cross-level interaction between plating condition and trait pathogen avoidance motivation. Specifically, we found significant differences in the slope of the relationship between trait pathogen avoidance motivation and TNF-*α* in the media only condition when compared with both the LPS (*B* = .28 (*SE* = .11), *t* = 2.42, *p* = .02) and PHA (*B* = .31 (*SE* = .12), *t* = 2.68, *p* = .01) plating conditions. We found no significant difference in the slope of trait pathogen avoidance motivation when comparing LPS and PHA conditions (*B* = .03, *p* = .11). Simple slopes tests revealed a significant negative slope of trait pathogen avoidance motivation in predicting TNF-α release for the media only condition (*B* = -.32 (*SE* = .11), *t* = -2.93, *p* = .005). For each unit increase in pathogen avoidance motivation, the model predicted a .32 unit decrease in TNF-α release. The slope of trait pathogen avoidance motivation was not significant in either the LPS condition (*B* = -.05, *p* = .29) or the PHA condition (*B* = -.01, *p* = .74).

These results conceptually replicated the pattern observed for IL-6 release. Higher trait levels of pathogen avoidance motivation predicted lesser TNF-α release in the absence of immune stimulation, but did not predict differences in the when an immune stimulus was present.

**Pathogen avoidance motivation and IL-1β**

The final model revealed significant main effects of plating condition on IL-1β release. IL-1β was higher in both the LPS condition (*B* = 2.82 (*SE* = .10), *t* = 26.88, *p* < .001) and in the PHA condition (*B* = 2.50 (*SE* = .11), *t* = 23.28, *p* < .001) than in the media only condition (by 2.82 units and 2.50 units, respectively). IL-1β release was also higher in the LPS condition than in the PHA condition by .32 units (*B* = .32 (*SE* = .02), *t* = 13.78, *p* < .001). The slope of time was significant, indicating that IL-1β release declined over time (*B* = -.01 (*SE* = .001), *t* = -10.75, *p* < .001). Trait levels of pathogen avoidance motivation did not significantly interact with the slope of time (*B* = .001, *p* = .34).

Unlike the results observed for IL-6 and TNF-α, no cross-level interaction between plating condition and trait pathogen avoidance motivation was found. Instead, results revealed only a main effect of trait pathogen avoidance motivation in the media condition (*B* = -.20 (*SE* = .09), *t* = -2.24, *p* = .03), indicating that each unit increase in pathogen avoidance motivation predicted a .20 unit reduction in IL-1β. This slope was not significantly different than those found in either the LPS (*B* = .10, *p* = .38) or PHA (*B* = .11, *p* = .33) plating conditions. Similar to what was observed with IL-6 and TNF-α, results revealed that trait pathogen avoidance motivation predicted decreased IL-1β release in the absence of an immune stimulus. Although the differences between the slopes of trait pathogen avoidance motivation were not significantly different in the spontaneous and stimulated cytokine release conditions, the negative slope in the media only condition was steeper than either stimulated release condition, conceptually replicating the results found for the other two cytokines.

**Alternative Models**

We ran additional HLM models that were analogous to our target model, except that perceived infectability (PI) was our predictor. As in our main model, PI was grand-mean centered and substituted for GA as a random effect in the final model for each cytokine outcome. Results of these alternative models revealed that the pattern observed for trait pathogen avoidance motivation was not repeated for PI, suggesting that pathogen avoidance motivation, per se, predicts reduced basal inflammation.

**Perceived Infectability and IL-6**

Results revealed one significant two-way, cross-level interaction between PI and plating condition on IL-6 release, indicating that the slope of PI was significantly different in the media only condition compared to the LPS condition (*B* = .24 (*SE* = .12), *t* = 2.01, *p* = .05) and marginally different from the PHA condition (*B* = .24 (*SE* = .12), *t* = 1.95, *p* = .06). No differences were found in the slope between the two mitogen conditions (*B* = .003 *p* = .89). Additionally, there was no significant interaction between PI and the slope of time (*B* = .00, *p* = .83). Simple slopes tests found that PI did not emerge significant as a predictor of IL-6 release in the media condition (*B* = -.22 *p* = .10), LPS condition (*B* = .02 *p* = .35), or PHA condition (*B* = .02 *p* = .37).

**Perceived Infectability and TNF-α**

Results revealed marginally significant differences between the slope of PI in the media only condition compared to LPS (*B* = .18 (*SE* = .10), *t* = 1.87, *p* = .07) and PHA (*B* = .17 (*SE* = .10), *t* = 1.77, *p* = .08). No significant difference was found in the slope of PI when comparing the LPS and PHA conditions (*B* = -.01, *p* = .76). Additionally, there was no significant interaction between PI and the slope of time (*B* = .00, *p* = .72). Furthermore, simple slopes tests found that PI did not significantly predict TNF-α release in the media condition (*B* = -.14, *p* = .17), LPS condition (*B* = .04, *p* = .22), or PHA condition (*B* = .04, *p* = .28).

**Perceived Infectability and IL-1β**

Assessing the impact of PI on the release of IL-1β revealed no significant interactions between PI and plating condition, indicating that the slope of PI was not significantly different when comparing the media condition to LPS (*B* = .12, *p* = .21) or to PHA (*B* = .12, *p* = .19), nor was the slope different between the two mitogen conditions (*B* = .01, *p* = .70). No significant interaction between PI and the slope of time was found (*B* = .00, *p* = .47). Simple slopes analysis revealed that PI did not predict release of IL-1β in the media condition (*B* = -.09, *p* = .23), LPS condition (*B* = .02, *p* = .61), or PHA condition (*B* = .03, *p* = .50).

Overall, one’s perceived vulnerability to illness produced a similar pattern of results as trait pathogen avoidance motivation, but no relationships between PI and cyotkine release reached significance. It is worth noting that the negative relationship between PI and spontaneous IL-6 release approached significance (*p* = .10), which is not unexpected given that the positive correlation between PI and GA was nearly significant (*p* = .11).

**References**

1. Derry HM, Fagundes CP, Andridge R, Glaser R, Malarkey WB, Kiecolt-Glaser J K. Lower subjective social status exaggerates interleukin-6 responses to a laboratory stressor. Psychoneuroendocrinology 2013;38:2676-2685.
2. Raudenbush SW, Bryk AS. Hierarchical linear models: Applications and data analysis methods. Vol. 1. Thousand Oaks (CA): SAGE Publications; 2002.
3. Muthén LK, Muthén BO. MPlus User’s Guide. 7th ed. Los Angeles: Muthén & Muthén, 1998-2012.
