# Supplementary material for: Behavioral immune system activity predicts downregulation of chronic basal inflammation
Source: PLoS One. 2018 Sep 20;13(9):e0203961. doi: 10.1371/journal.pone.0203961 (PMC6147464; doi:10.1371/journal.pone.0203961)
Supplement: S1 Table — All participants were non-smokers and came in fasting and healthy (i.e., no illnesses reported two weeks prior to the testing session). Participants were instructed not to consume alcohol, exercise, or take anti-inflammatory medications for two days prior to their testing session. All women in the sample were non-pregnant and not on hormonal contraceptives. Women’s sessions all took place 4–7 days after the start of their menstrual cycles. (DOCX) [file pone.0203961.s002.docx]

**Table S1.** **Characteristics of the Sample for Study 1 (*N* = 62)**.

| Sex: Men = 38; Women = 24  Age (18–24): *M* = 18.97, *SD* = 1.28  Race  White: 87.10% (*n* = 54)  Black: 1.61% (*n* = 1)  Hispanic: 6.45% (*n* = 4)  Asian: 3.23% (*n* = 2)  Multiracial: 1.61% (*n* = 1)  Parental education (1–9; 1 = Some high school; 9 = Doctoral or law degree)  Mother: *M* = 6.00, *SD* = 1.58  Father: *M* = 6.15, *SD* = 2.03  Parent alive  Mother: Yes = 61; No = 0; No Response = 1  Father: Yes = 60; No = 2  Body mass index (17.5–29.6): *M* = 22.52 kg/m^2^, *SD* = 2.68  Typical activity level (1–7; 1 = Light; 7 = Strenuous): *M* = 4.18, *SD* = 1.35  Hours of sleep previous night: *M* = 7.19 hrs., *SD* = .92  Childhood SES (1–7): *M* = 4.95, *SD* = 1.18 |
| --- |

*Note.* All participants were non-smokers and came in fasting and healthy (i.e., no illnesses reported two weeks prior to the testing session). Participants were instructed not to consume alcohol, exercise, or take anti-inflammatory medications for two days prior to their testing session. All women in the sample were non-pregnant and not on hormonal contraceptives. Women’s sessions all took place 4–8 days after the start of their menstrual cycles.
