# Supplementary material for: Behavioral immune system activity predicts downregulation of chronic basal inflammation
Source: PLoS One. 2018 Sep 20;13(9):e0203961. doi: 10.1371/journal.pone.0203961 (PMC6147464; doi:10.1371/journal.pone.0203961)
Supplement: S2 Table — (DOCX) [file pone.0203961.s003.docx]

**Table S2. Characteristics of the Sample for Study 2 (*N* = 193).**

Pregnancy Status

Non-Pregnant: *n* = 102

Pregnant: *n* = 91

Age: *M* = 28.56 years, *SD* = 5.59

Relationship Status

In a relationship: *n* = 162

Single: *n* = 29

Race

White/Caucasian: 71.5% (*n* = 138)

Black/African American: 16.6% (*n* = 32)

Other: 11.9% (*n* = 23)

Childhood SES (1-7): *M* = 4.16, *SD* = 1.57

Current SES (1-7): *M* = 4.58, *SD* = 1.31

Number of children: *M* = 0.54, *SD* = 0.95
