# Supplementary material for: Behavioral immune system activity predicts downregulation of chronic basal inflammation
Source: PLoS One. 2018 Sep 20;13(9):e0203961. doi: 10.1371/journal.pone.0203961 (PMC6147464; doi:10.1371/journal.pone.0203961)
Supplement: S1 Fig — Associations between GA and oxidative stress in Study 2 for control women (panel A) and pregnant women (panel B). Pregnancy status did not moderate the significant relationship between trait pathogen avoidance motivation and oxidative stress. (DOCX) [file pone.0203961.s004.docx]

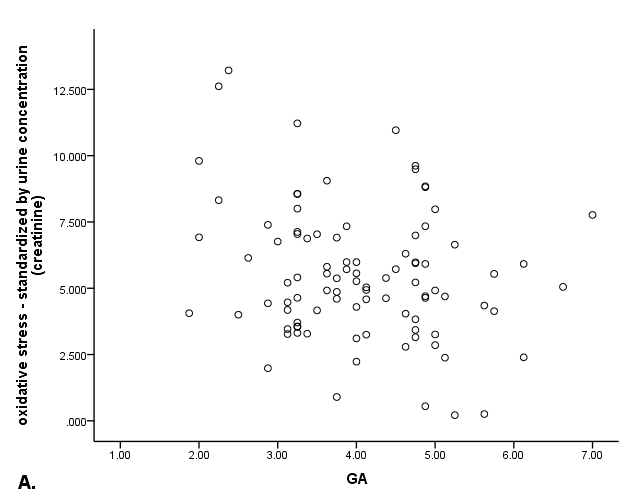


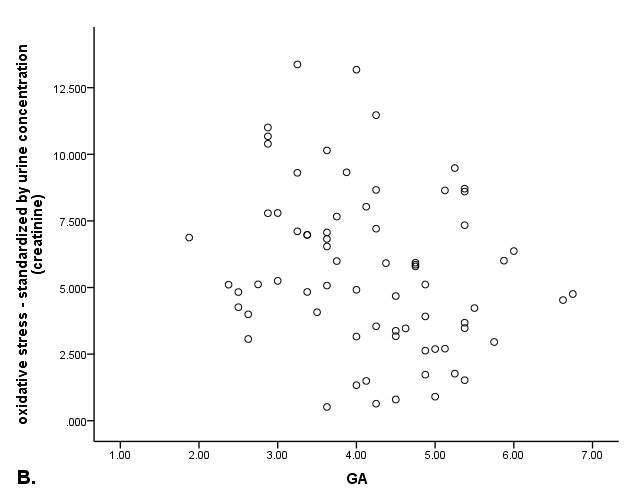


**Fig. S1.** **Associations between GA and oxidative stress in Study 2 for control women (panel A) and pregnant women (panel B).** Pregnancy status did not moderate the significant relationship between trait pathogen avoidance motivation and oxidative stress
